# Assessment of the efficacy of field and laboratory methods for the detection of *Tropilaelaps* spp.

**DOI:** 10.1371/journal.pone.0301880

**Published:** 2024-09-06

**Authors:** Maggie C. Gill, Bajaree Chuttong, Paul Davies, Dan Etheridge, Lakkhika Panyaraksa, Victoria Tomkies, George Tonge, Giles E. Budge

**Affiliations:** 1 Department for Environment Food and Rural Affairs, Horizon House, Bristol, United Kingdom; 2 Department of Entomology and Plant Pathology, Meliponini and Apini Research Laboratory, Faculty of Agriculture, Chiang Mai University, Chiang Mai, Thailand; 3 National Bee Unit, Animal Plant Health Agency, York Biotech Campus, Sand Hutton, York, United Kingdom; 4 Fera Science Ltd, York Biotech Campus, Sand Hutton, York, United Kingdom; 5 School of Natural and Environmental Sciences, Newcastle University, Newcastle upon Tyne, United Kingdom; University of Alberta, CANADA

## Abstract

*Tropilaelaps* spp. are invasive mites that cause severe disease in *Apis mellifera* colonies. The UK has deployed an elaborate surveillance system that seeks to detect these mites early in any invasion to allow the best opportunity to eradicate any incursion. Effective field and laboratory protocols, capable of reliably detecting low numbers of mites, are key to the success of any intervention. Here we compared the efficacy of established field monitoring using brood removal with an uncapping fork, and brood ‘bump’ methods with novel methods for *Tropilaelaps* detection modified from *Varroa* monitoring schemes. In addition, we monitored the efficacy of the laboratory method for screening for mites in hive debris by floating mites in ethanol. Our results clearly indicated that novel methods such as uncapping infested brood with tweezers, catching mite drop using sticky traps and rolling adult bees in icing sugar were all significantly more likely to detect *Tropilaelaps* than existing methods such as using an uncapping fork on infested brood, or the brood ‘bump’ method. Existing laboratory protocols that sieved hive debris and then floated the mite containing layer failed to detect *Tropilaelaps* mites and new efficacious protocols were developed. Our results demonstrated that the national surveillance protocols for *Tropilaelaps* mite detection required modification to improve the early detection of this damaging invasive mite.

## Introduction

*Tropilaelaps* mites are native brood parasites of the Asian honey bee species *Apis dorsata*, *Apis breviligula* and *Apis laboriosa* which have successfully jumped host to introduced colonies of *Apis mellifera*. The term *Tropilaelaps* mites refers to the four currently identified species of *Tropilaelaps*, *T*. *mercedesae*, *T*. *clareae*, *T*. *thaii* and *T*. *koenigerum*, of these *T*. *mercedesae* and *T*. *clareae* are known to infest *A*. *mellifera*. The current range of *Tropilaelaps* mites is not fully characterised [1], but until recently it was thought their spread restricted these mites to large areas of Asia, bordering onto Europe [2, 3]. However, Namin, *et al*. (2024) [4] and Brandorf, *et al*, (2024) [5] have recently confirmed the presence of *T*. *mercedesae* on *A*. *mellifera* in the Krasnador and Rostov-on-don regions of Russia, thus confirming its presence in Europe. This recent finding confirms *T*. *mercedesae* is an emerging global threat to *A*. *mellifera* that has the potential to parallel the near-global spread of *Varroa destructor* [6–8].

*Tropilaelaps* spp. reproduce within the capped brood of their hosts but have a shorter phoretic phase plus, a faster rate of reproduction than *Varroa* and have the ability to mate outside brood cells [9]. These life history traits allow populations of *Tropilaelaps* spp. to increase more rapidly than *Varroa* [10]. Brood feeding causes physical damage [9] and *Tropilaelaps* mites are known to transmit viruses which have the potential to kill or shorten the life of the developing bee [11–14]. Natural behaviours such as frequent absconding, migration, and high levels of grooming and hygienic behaviour of the natural Asian honey bee hosts play an important role in the control of *Tropilaelaps* spp. [15, 16], resulting in low infestation levels and concomitant low colony mortality [17]. *A*. *mellifera* has not yet evolved a symbiotic parasite-host relationship, resulting in high mite levels and high rates of colony mortality [18].

The presence of brood is thought to be a prerequisite for *Tropilaelaps* mites to survive within an *A*. *mellifera* colony, with *T*. *clareae* demonstrating survival of only three days in artificial conditions [19, 20]. There is suspicion that, under natural conditions, *Tropilaelaps* spp. could survive for longer periods in the absence of brood particularly as its natural host *A*. *dorsata* regularly exhibit extended broodless periods during their seasonal migrations [1, 21, 22], and *T*. *mercedesae* has been shown to overwinter in *A*. *mellifera* colonies in temperate climates where brood is often absent [5, 23].

Increased global trade and changes in beekeeping practices provide transmission routes for *Tropilaelaps* spp. which include live honey bee sales, migratory beekeeping and the potential distribution of feral colonies or swarms on shipping containers and cargo vessels [24].*Tropilaelaps* spp. are statutory notifiable pests in most countries worldwide because of a combination of likely global distribution coupled with significant economic losses because of high mortality rates in *A*. *mellifera* colonies [25]. The National Bee Unit (NBU) is a government body responsible for the surveillance of honey bee (*A*. *mellifera*) colonies in England and Wales for the presence of the statutory notifiable pests Small Hive Beetle (*Aethina tumida*) and *Tropilaelaps* spp. [26]. The early interception of exotic pest incursions is imperative to increase the chances for the successful eradication of invasive pest species [27]. The UK operates an extensive monitoring programme for *Tropilaelaps* spp. that includes exotic pest risk points that are designated using a risk based classification system that is based on the work of Keeling, *et al*. (2017) [27], with ports and queen importers identified as the highest incursion risk of an exotic pest incursion. A network of enhanced sentinel apiaries (ESA) positioned in close proximity to the exotic pest risk points where bee inspectors from the NBU carry out colony inspections specifically targeted at the detection of *Tropilaelaps* spp. ESAs are complemented by voluntary sentinel apiaries (VSA), where volunteer beekeepers close to an exotic pest risk point regularly submit floor debris samples for laboratory analysis for *Tropilaelaps* spp.

The NBU operate a standard operating procedure (SOP) for the detection of *Tropilaelaps* spp. in sentinel apiaries (ESA and VSA) adapted from techniques described by Pettis, *et al*. (2013) [28]. The three surveillance techniques include the removal and inspection of 200 cells of sealed brood using an uncapping fork, the ‘bump method’, where all brood frames are hit against a solid surface to dislodge mites onto a white monitoring tray and the laboratory screening of hive floor debris samples for *Tropilaelaps* spp. (and *A*. *tumida*). Should *Tropilaelaps* be detected using the above three methods, then enhanced surveillance would be completed on colonies within 16 km of the outbreak using a miticide in conjunction with a sticky floor insert. It is worth noting that beekeepers often object to the damage caused to brood by the bump method as well as the destructive nature of brood uncapping.

The aim of this study was to determine the efficacy of the existing field and laboratory protocols employed for the detection of *Tropilaelaps* spp. as part of the national exotic pest surveillance scheme and to test novel methods for mite detection to improve the chance of national surveillance systems to detect these damaging mites early in any invasion.

## Methods

### Field inspections

**Fig 1:**
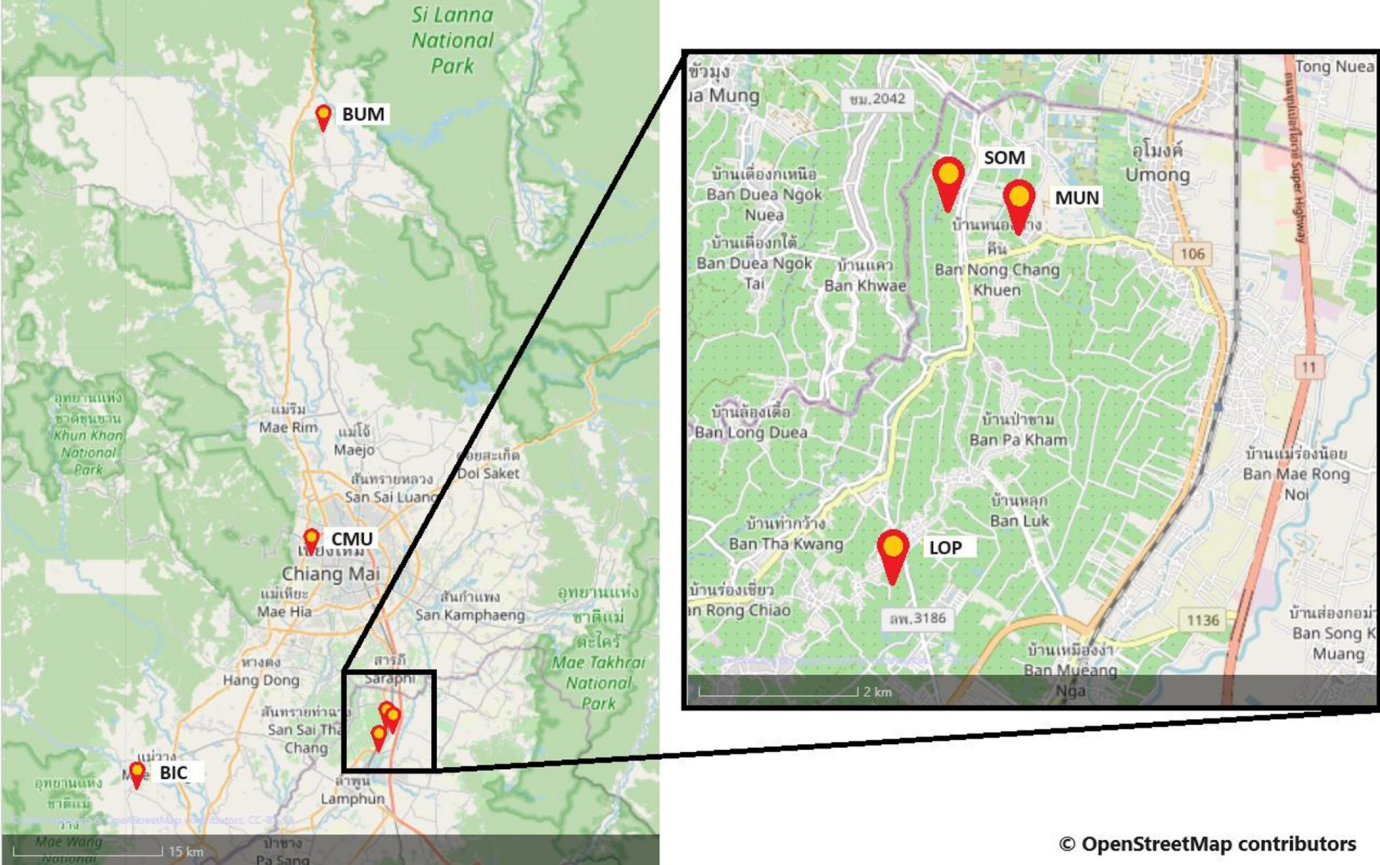
Map of apiary locations in Thailand. Contains information from OpenStreetMap and OpenStreetMap Foundation, which is made available under the Open Database License. [30].

In total, 60 colonies of *A*. *mellifera* were sourced from six apiaries in Chiang Mai province Thailand (Fig 1). The relevant permits were secured by Chiang Mai University from DOA Thailand prior to this research. Each colony was assessed for strength with a visual examination of the colony and the number of frames of bees and brood present were recorded. Each colony was then assessed using all four of the field protocols detailed below. Only one method for monitoring mite drop could be deployed on each colony, and so colonies were randomly allocated to receive either oil or sticky inserts, as both these media are known to trap mites [29]. Beekeepers were asked to not treat colonies for mites for at least two brood cycles prior to assessment, but colony management was not controlled prior to this point. Multiple detection methods were trialled, and the numbers of *T*. *mercedesae* were counted for each method on each colony.

### Field protocols

#### Brood uncapping

The SOP for the detection of *Tropilaelaps* spp. specified that 200 sealed drone brood cells containing pink eyed pupae should be uncapped using an uncapping fork, however brood of the correct age were rarely present in the sampled colonies. When carried out mites became trapped by exudate from the damaged pupae or were difficult to identify amongst the exudate from the damaged pupae. As such, the method was modified to sample a 10 x 10 rhombus template of 100 sealed brood cells. In line with methods described by Pettis, *et al*. (2013) [28] and Dietemann, *et al*. (2015) [31] fine-nosed forceps were used to remove the immature honey bee from each cell and the larva or pupae were carefully inspected for mites to count the number of mature and immature mites within cells. If brood from the final larval instar (day 9) to the pharate pupal stage (day 13) or brood that was post pink eyed stage (day 18) were removed, then *Tropilaelaps* mites tended to not be found on the pupae, but instead remained in the cells. They could be dispersed by gently blowing over the brood or by using a fine artist brush dipped in honey to sweep the cells. Typically, *Tropilaelaps* mites would be seen on extracted pupae that were between 14 to 17 days old and these observations were in line with those made by Han, *et al*. (2024) [10]. A headtorch and an illuminated 40x magnification hand lens was also employed to aid mite discovery.

#### Comb bump method

**Fig 2:**
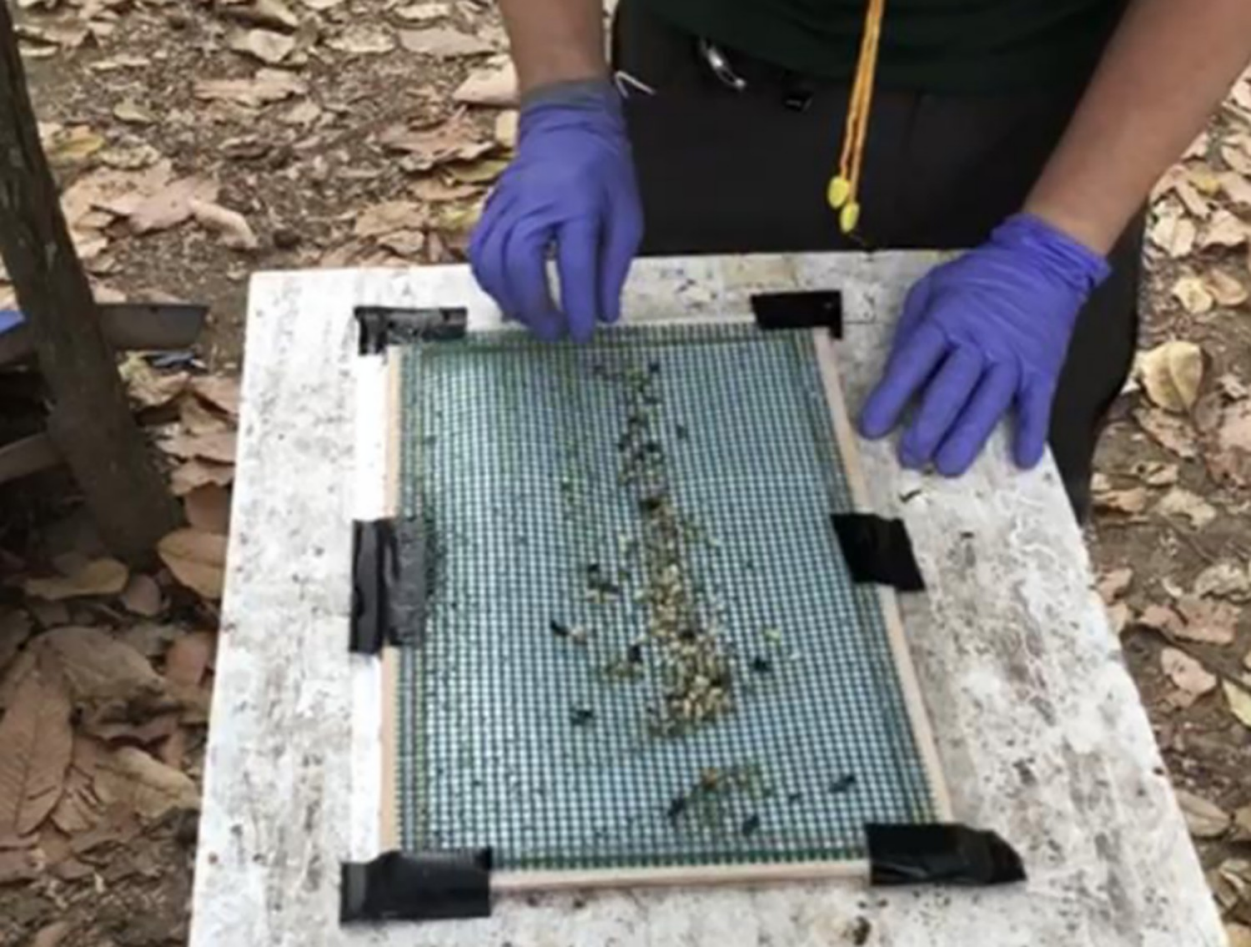
Dead brood on a sticky floor insert placed below a mesh screen from a colony where brood frames had been ‘bumped’ 24 hours previously.

The current surveillance procedure was adapted from the bump method described by Pettis, *et al*. (2013) [28]. The SOP originally specified that adult bees were shaken from every brood frame and then each brood frame was held horizontally over a white tray with the wooden edge of the frame firmly hit twice per side on the edge of the tray allowing mites to fall into the tray. Beekeepers expressed concerns about the damage this caused the brood (Fig 2), therefore this method was refined, and adult bees were removed from one sealed and one unsealed brood frame selected at random. This offered a compromise to beekeepers where the majority of the brood was still bumped while sampling a representation of both sealed and unsealed brood.

Each frame was held horizontally over a white tray with the wooden edge of the frame firmly hit four times per side on the edge of the tray allowing mites to fall into the tray. The tray was then examined for the presence of dislodged phoretic mites with a hand lens (x40) and head torch.

#### Hive debris

UK beekeepers generally use open mesh floors which allow for the easy collection of hive debris via a removable sliding tray placed below the mesh floor. Debris samples collected in such a manner are routinely collected from colonies and sent to the laboratory for analysis following a set protocol. Thailand beekeepers use an adapted Langstroth hive where hive floors are attached to the main hive brood body. As such, all the brood frames had to be removed to access the floor debris and samples were scraped from the hive floor and bagged in ziplock bags for later laboratory analysis. Laboratory mite surveillance utilised methods for the detection of *Tropilaelaps* spp. in floor debris samples outlined by Pettis, *et al*. (2013) [28], Dietemann, *et al*. (2015) [31] and the OIE Terrestrial Manual (2008) [32]. To assess the presence of mites, Endecott sieves were stacked on top of each other in decreasing size (1.7 mm, 1 mm, 0.7 mm and 0.355 mm) and hive debris placed in the top sieve. The sample was then flushed through the sieves using tap water passed through a small shower head, resulting in size separation of the debris. The smallest sieve (0.355 mm) is fine enough to retain any *Tropilaelaps* mites present in the sample and the contents were placed in a small washing up bowl of tap water, (volume added alters according to the diameter of the bowl, but the depth should be 2cm) allowing mites to float after it was established that mites did not reliably float in ethanol (≥98%). The sample was assessed in good light, using either a large magnifying glass or a jeweller’s hand lens (x40), however any *Tropilaelaps* mites present in samples could not be reliably identified from amongst the hive debris as the debris also floated in the water. Therefore, ethanol flotation was discounted as a detection technique early on in this study.

In the UK, sticky inserts would normally be deployed on the removable sliding tray of open mesh floors, but the configuration of Thai hives meant that floor inserts had to be modified slightly. Floor inserts were made up of a shallow wooden frame measuring 381 mm x 534 mm which supported a plastic mesh with a 4 mm hole size. This mesh frame was then inserted to cover the floor of the study hives. A sticky insert (Vita Europe, commercially available in North America and Europe, but not the UK) was used in half the colonies and a laminated card coated with a thin layer of vegetable oil was used in the remaining colonies, with the mesh frame being used to stop the bees from accessing both types of floor insert. Beekeepers will often coat floor inserts with substances such as vegetable oil or petroleum jelly as a method of monitoring *Varroa* mite drop in colonies. Both methods were used in this study to assess if ‘home made’ monitoring offered an effective alternative to commercially available (but difficult to source) sticky inserts which could be required if *Tropilaelaps* spp. were ever detected in the UK. No UK approved acaricide strips were used to promote mite drop as specified in the SOP as experimental permits could not be obtained. As such, only natural mite drop was recorded. Floor inserts were inserted for 24 hours, removed carefully and the mite count completed in the laboratory.

#### Monitoring mites on adult bees

The frame containing the queen was found and set to one side. The adult bees from one frame of mostly open brood and one frame of mostly closed brood were shaken into a smooth-sided bowl. Approximately 300 adult bees were scooped into each of three *Varroa* EasyCheck mite counters (Veto Pharma). The first counter was treated with 300 ml ethanol (≥98%) to dislodge adult phoretic mites, and the mites counted through the clear base after one minute of swirling, essentially as described by Pettis, *et al*. (2013) [28]. This is commonly referred to be beekeepers as an alcohol wash and shall hence forth be referred to as alcohol wash. The second counter was treated with 25 g of icing sugar before the bees were rolled for one minute as described by Dietemann, *et al*. (2015) [31]. The *Varroa* EasyCheck counter was then left to stand out of direct sunlight for one minute before the icing sugar was shaken out onto a white tray and assessed for the presence of mites. This process was repeated after a further two minutes to allow the bees further opportunity to groom. The third counter was treated with a steady supply of CO_2_ until the bees were fully anaesthetised. The bees were then gently rolled for fifteen seconds, before being shaken over a white tray and checked for the presence of mites using a hand lens (x40) and headtorch. Fresh ethanol and icing sugar was used for each trial and the *Varroa* EasyChecks were washed, dried and thoroughly examined for mites in between trials.

### Statistical analyses

To prevent bias towards monitoring method due to variation in mite levels between colonies, colonies were only included where data were gathered for all monitoring methods. All models were implemented using R version 4.2.3 [33]. First, we used a binomial generalised linear model (GLM) with a logit link function, with the presence of mites as a response variable and monitoring method and apiary as the independent variables. Second, we used zero-inflated regression models for count data via maximum likelihood implemented in the *pcsl* package [34], with the number of mites as the response variable and monitoring method and apiary as the independent variables. Model fit was compared using the Akaike Information Criterion (AIC) and 95% confidence intervals of the estimates were calculated using the coefficients and standard error from the GLM summary table.

The data were partitioned into three different sets to account for the fact that half the colonies had mite drop monitored using oil, and the other half used sticky traps. First, the method of mite drop was removed to retain the highest number of observations for the remaining monitoring treatments. Second, the data were filtered to only those colonies with data using oil to monitor mite drop, and the third partition contained only those colonies with data using sticky traps to monitor mite drop. The efficacy of the monitoring methods was expressed as the odds ratio of detecting mites compared to the alcohol wash of 300 adult bees with ethanol, because this was the standard detection method for England and Wales prior to this study.

## Results

**Fig 3:**
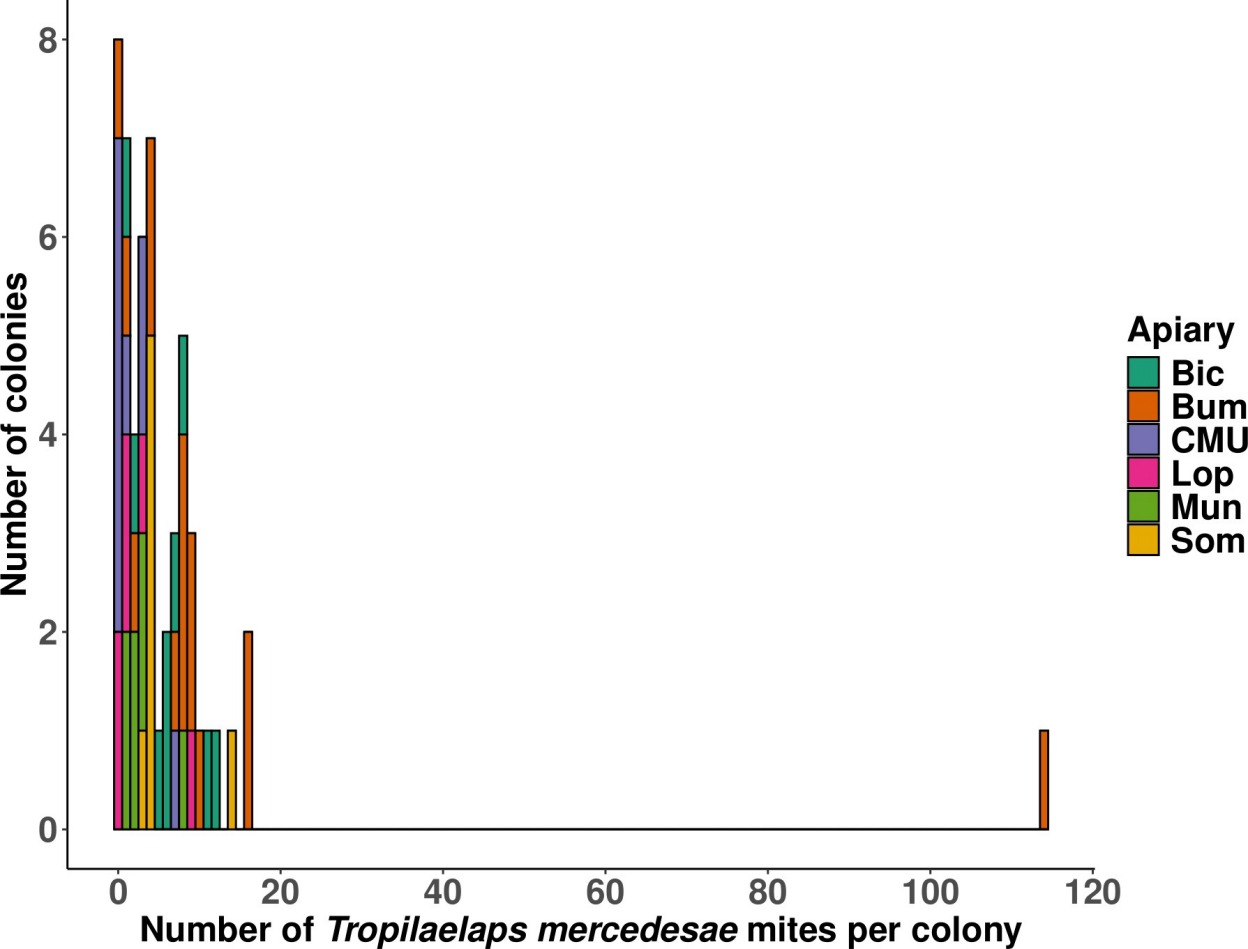
The number of *T*. *mercedesae* observed mites per colony and by apiary.

Of the 60 colonies that were examined, colony size averaged 5.3 frames of bees (±5.1) and 3.8 (±2.7) frames of brood. *T*. *mercedesae* was detected in 80% of colonies and 100% of apiaries (Fig 3). In total, seven colonies were removed from the analysis because the results from one or more monitoring method was absent. Of these seven colonies, five were deemed too small to provide the necessary brood samples for monitoring, and the uncapping method was incorrectly deployed in two colonies. In total, 53 colonies were assessed for the efficacy of alcohol wash, brood bump, CO_2_, uncapping and sugar roll. Mite drop was monitored using oil in 24 colonies and using sticky traps in 29 colonies. Ethanol floatation was not used on floor debris samples because preliminary work established that *Tropilaelaps* spp. do not reliably float in ethanol thus this technique was deemed ineffective.

### Comparing the ability of each method to detect *Tropilaelaps* mites

The AIC was consistently lower for the binomial GLM containing treatment and apiary (All colonies = 278.55; Oil drop only = 160.52; Sticky drop only = 193.24), compared to treatment alone (All colonies = 301.78; Oil drop only = 180.67; Sticky drop only = 203.61), and so this was chosen as our final model. Method of mite detection had a significant effect on the presence of *Tropilaelaps* mites in the colony for all three analyses (Table 1).

**Table 1:** Summary of binomial GLMs comparing *T*. *mercedesae* detection using all methods in the absence of mite drop (All colonies; n = 53); including only those colonies that received oil to monitor mite drop (Oil drop only; n = 24); and including only those colonies that received sticky traps to monitor mite drop (Sticky drop only; n = 29). The Intercept represents the alcohol wash for apiary location BIC.

All colonies	Method/apiary	Estimate	St.error	Z value	P value
	(Intercept)	-1.11	0.469	-2.37	<0.05
*Method*	Bump	0.23	0.477	0.48	NS
	CO_2_	-0.71	0.542	-1.3	NS
	Uncapping	2.79	0.516	5.41	<0.001
	Sugar roll	1.47	0.464	3.17	<0.01
*Apiary name*	Bum	0.60	0.445	1.36	NS
	CMU	-1.85	0.597	-3.09	<0.01
	Lop	-1.46	0.640	-2.28	<0.05
	Mun	-0.64	0.557	-1.16	NS
	Som	0.35	0.529	0.66	NS
**Oil drop only**	(Intercept)	-1.08	0.662	-1.63	NS
*Method*	Bump	0	0.703	0	NS
	CO_2_	-1.22	0.818	-1.49	NS
	Uncapping	2.34	0.753	3.11	<0.01
	Oil drop	0.24	0.694	0.35	NS
	Sugar roll	1.36	0.698	1.95	??
*Apiary name*	Bum	1.27	0.605	2.1	<0.05
	CMU	-2.29	0.935	-2.45	<0.05
	Lop	-0.57	0.765	-0.75	NS
	Mun	-0.95	0.799	-1.18	NS
	Som	0.66	0.719	0.91	NS
**Sticky drop only**	(Intercept)	-1.6	0.645	-2.47	<0.05
*Method*	Bump	0.45	0.679	0.67	NS
	CO_2_	-0.28	0.749	-0.37	NS
	Uncapping	3.4	0.75	4.53	<0.001
	Sticky drop	1.51	0.65	2.32	<0.05
	Sugar roll	1.67	0.651	2.57	<0.05
*Apiary name*	Bum	0.32	0.55	0.59	NS
	CMU	-1.33	0.694	-1.92	NS
	Lop	-2.22	0.961	-2.31	<0.05
	Mun	0.32	0.644	0.5	NS
	Som	0.77	0.642	1.19	NS

**Fig 4:**
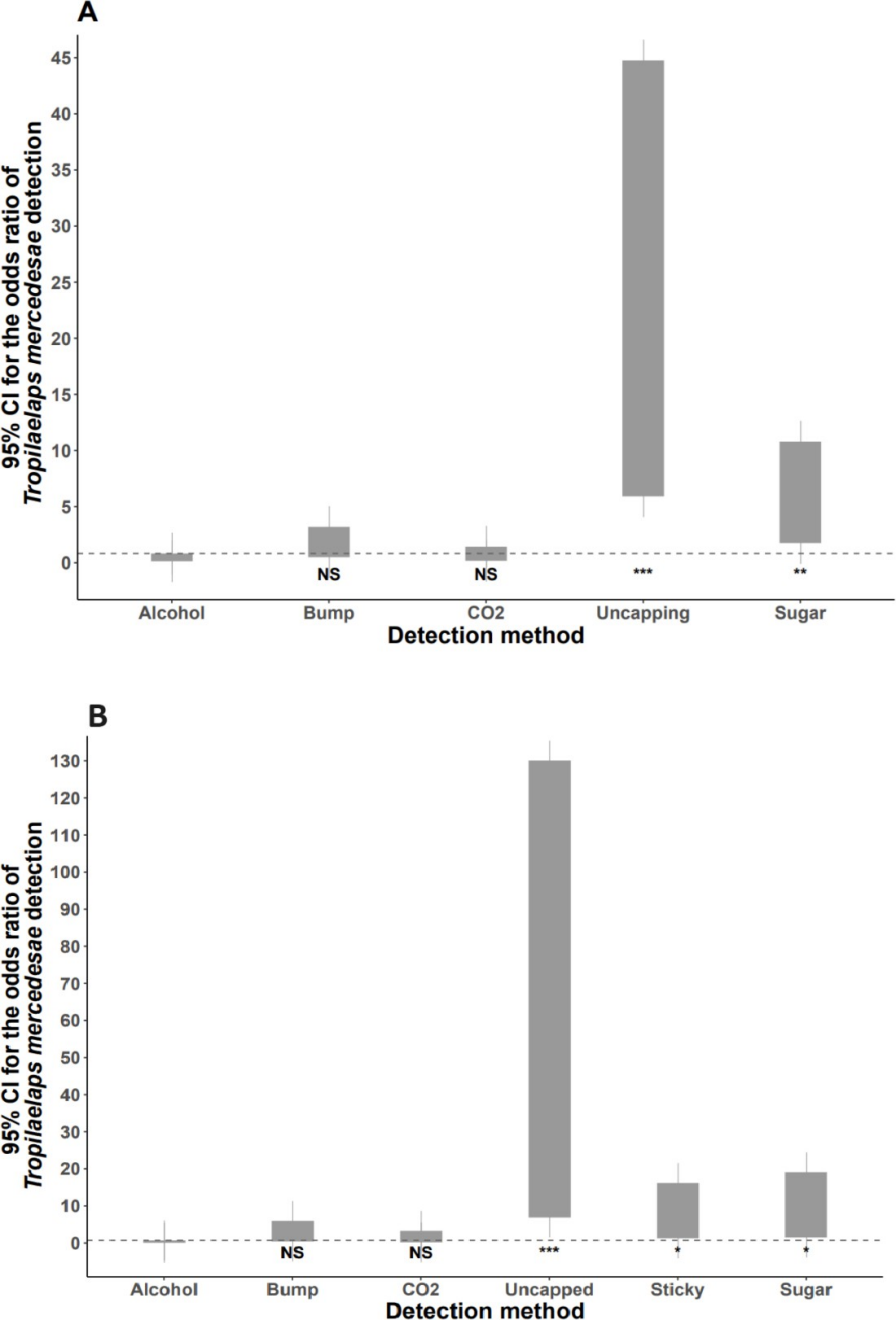
The odds ratio of *T*. *mercedesae* detection using different methods when compared to alcohol washing mites from ~300 adult bees. The grey bars represent the uncertainty (95% confidence intervals) around the estimated odd ratios. The dashed horizontal line represents the upper 95% CI of the estimate for alcohol. A) All colonies excluding mite drop protocols (n = 53); B) Data restricted to only those colonies where mite drop was monitored using sticky traps (n = 24).

When comparing detection methods across all 53 colonies, it was clear that both uncapping brood and a sugar roll of bees were more likely to detect *Tropilaelaps* mites than an alcohol wash of adult bees in ethanol. Bumping the brood and using CO_2_ to knock down the mites on adult bees were not better than alcohol wash (Table 1; Fig 4A). When restricting to those colonies where mite drop was monitored using sticky traps, the sticky traps were significantly more likely to detect the presence of *Tropilaelaps* mites when compared to an alcohol wash of adult bees (Table 1; Fig 4B).

### Comparing the ability of each method to detect the number of *Tropilaelaps* mites

**Fig 5:**
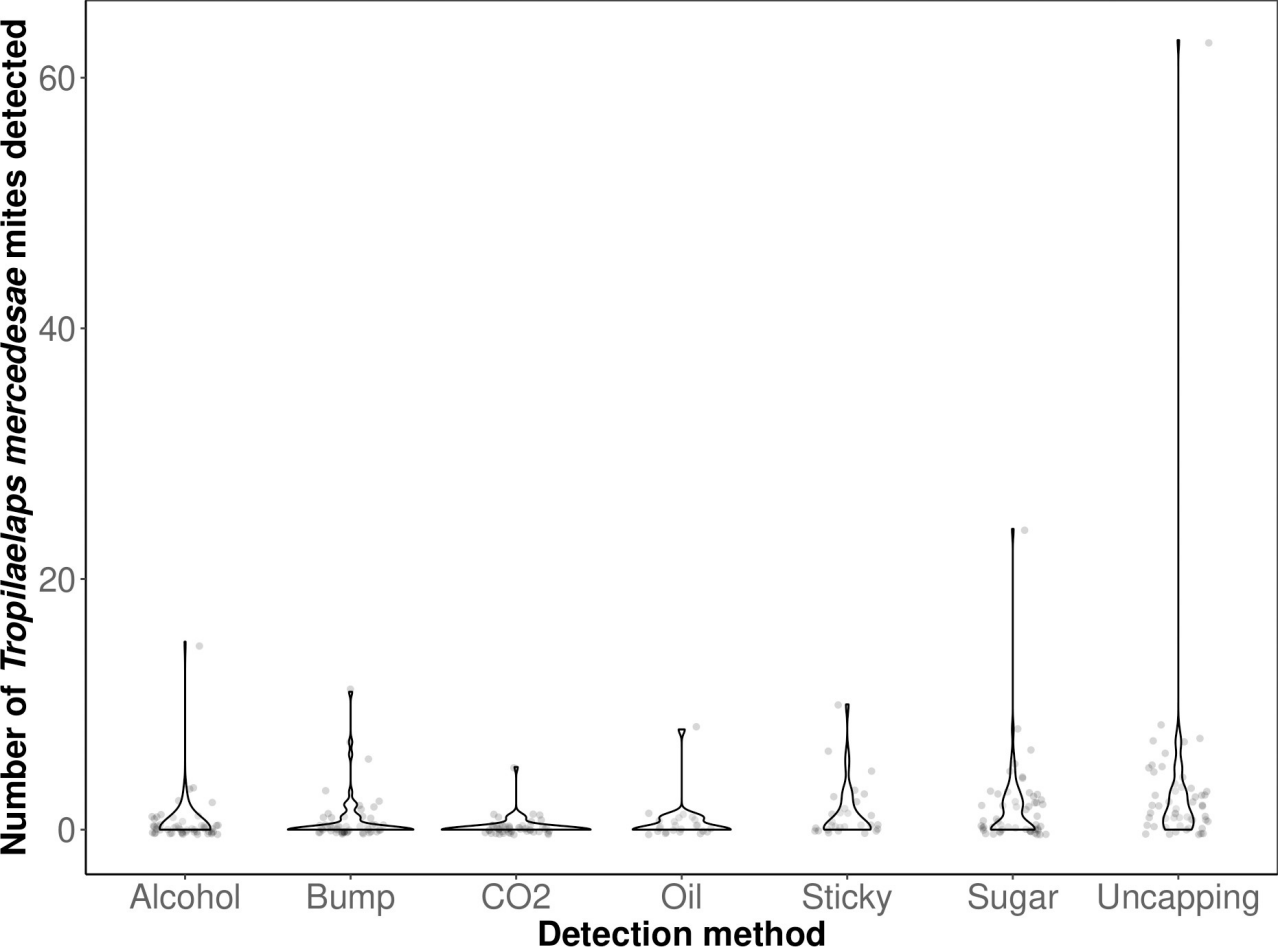
Violin plot of the *T*. *mercedesae* count data for all colonies to show all data points and the solid outline highlights the data distribution.

Despite efforts to transform the data and to run negative binomial models, the quantitative mite data were not amenable to analysis because of a high number of zeros and then a large span of values for those methods testing positive for mites. The spread of the data are shown (Fig 5) and the raw data are provided (see S1 File).

## Discussion

Our study has refined the *Tropilaelaps* mite detection protocols for deployment in England and Wales for the purposes of exotic pest surveillance. We established that whilst the use of an uncapping fork is appropriate for the detection of *Varroa* mites [31], this technique was not suitable for the detection of *Tropilaelaps* due to their size, levels of mobility and fragility. The use of tweezers to remove wax cappings and extract brood was shown to be the most effective detection method for *Tropilaelaps* mites. The selection of post-larval brood was unpredictable as there is no reliable way to determine the developmental stage of brood in sealed cells. Therefore, the technique for assessing if *Tropilaelaps* mites were present in an uncapped cell did need minor adaptation depending on the development stage of the brood removed from each cell. The use of an icing sugar roll of 300 adult bees, which is a standard method for *Varroa* spp. detection [31], was also an effective and novel method to detect phoretic *Tropilaelaps* mites in a honey bee colony. This offers a non-destructive, quick, cheap, and easily deployable detection method that has the potential to be utilised not only during government surveillance but also by beekeepers themselves. Both brood uncapping using tweezers and an icing sugar roll of adult bees offer reliable *Tropilaelaps* detection techniques, and as such the most field appropriate method can be selected. Whilst uncapping cells using tweezers is the more reliable of the two methods for detection of *Tropilaelaps* mites it is time consuming, and an icing sugar roll offers a quick, reliable non-destructive technique that is more palatable to beekeepers.

An alcohol wash [31], and CO_2_ monitoring of adult bees in *Varroa* EasyChecks proved ineffective at reliably detecting *Tropilaelaps* mites. Although not definitively demonstrated during this work, it is our belief that the smaller size of *Tropilaelaps* combined with relatively long legs when compared to *Varroa*, meant that *Tropilaelaps* mites became entangled with the adult bees and were too small to be dislodged into the collection portion of the *Varroa* EasyChecks. However, the use of icing sugar clogged the *Tropilaelaps* mite legs (mites were observed on their backs struggling to remove icing sugar from between their legs) and caused the adult bees to groom dislodging mites and allowing them to enter the collection portion of the *Varroa* EasyChecks.

Natural mite drop in conjunction with sticky floors can be reliably used to detect *Tropilaelaps*. Variable light conditions and the need to wear veils made examination of floor inserts in the field for detecting *Tropilaelaps* mites difficult and instead laboratory analysis with good lighting and magnification was needed to conclusively determine if *Tropilaelaps* were present in samples. The screening of floor debris via sieving and ethanol flotation was shown to be ineffective for the detection of *Tropilaelaps* mites present in samples. Mites became damaged by the hive debris collection process or entangled in clumps of hive debris making them unrecognisable and the sieving process ineffective. Mites that were dislodged by sieving could not be made to reliably float in 96% ethanol. These issues had previously not been identified by other research [6, 28, 35] and this work has resulted in adaptation and amendments being made to all in field and laboratory *Tropilaelaps* surveillance and screening protocols in England and Wales.

An important consideration for anyone assessing the efficacy of field and laboratory methods for the detection of *Tropilaelaps* spp. is the level at which a novel infestation needs to be detected to offer the best possible chance for eradication of a pest. Our study measured the efficiency of *T*. *mercedesae* detection techniques in Thailand in February, when *T*. *mercedesae* levels were low with a sum of between zero and twenty mites detected per colony for all methods (Fig 3). Our results are likely to be particularly applicable for use in an outbreak situation, where levels of mites are low. It could be that some methods that performed poorly in our study, like the bump method and an alcohol wash, might show improved performance in colonies with higher levels of mites, but this would need further study. Our results provide a level of reassurance about the effectiveness of *T*. *mercedesae* detection techniques in colonies with low levels mites, which is appropriate for statutory monitoring programmes that hope to detect new incursions.

Symptoms of infestation of *Tropilaelaps* mites closely resemble that of *Varroa* and given that beekeepers sometimes struggle to detect *Varroa* mites [9], detection by beekeepers seems unlikely. The small size and high mobility of *Tropilaelaps* mites combined with their potential to survive in broodless scenarios increases its potential for transmission around the globe [23]. Thus proactive, targeted and robust surveillance is crucial to prevent its colonisation of new global areas. Recent novel infestations of *V*. *destructor* in countries such as La Reunion Island and Australia have demonstrated that the introduction of a single mite to a previously *Varroa* free area led to colony losses as high as 64% in some areas during the year following introduction [36, 37]. It is reasonable to extrapolate that similar colony losses could be expected if *Tropilaelaps* spp. were introduced to the UK at low levels [38] especially as both Namin, *et al*. (2024) and Brandorf, *et al*. (2024) [4, 5] reported that beekeepers experienced high colony losses where *Tropilaelaps* has been recently detected for the first time. Given the limited research and data available on *Tropilaelaps* spp. when compared to other exotic pests such as small hive beetle and *Varroa*, the difficulties associated with detecting *Tropilaelaps*, the similarities in symptomology to a *V*. *destructor* infestation and the potential high levels of colony mortality a novel colonisation would cause it is crucial that government agencies and beekeepers around the world remain vigilant for this potentially devastating pest.

Our work highlights the importance of testing surveillance procedures for invasive pests in ‘real-life’ inspection scenarios. While previous studies developed detection techniques such as the bump method and brood examination, the practicality and appetite of beekeepers to participate in surveillance using certain techniques needs to be an important consideration. It should not be assumed that surveillance methods will be appropriate for incursions when techniques have been developed in experimental conditions. If the findings of this research are used to advise surveillance procedures, a balance needs to be struck between the use of the significantly more robust detection of *Tropilaelaps* using uncapping with the sacrifice of 100 brood cells versus the non-destructive and more rapid detection of phoretic mites using icing sugar. Whilst the differences in beekeeping husbandry and climate between the UK and Thailand are significant, we assume these techniques would be easily transferred to the UK. Honey bee behaviour and brood development are comparable between the two countries and factors such as husbandry and climate have limited impact on the *Tropilaelaps / A*. *mellifera* interactions. This is demonstrated by the ability of *Tropilaelaps* mites to spread to new regions with diverse climates and differing beekeeping practices [4, 5, 27].

The use of an alcohol wash or CO_2_ monitoring of adult bees for *Tropilaelaps* mites were rejected as unreliable detection methods. The use of the bump method as part of the standard operating procedure for *Tropilaelaps* mite detection in the UK has now been discontinued based on the results of this study as it was demonstrated to be ineffective and destructive. When colonies were examined 24 hours after the bump method had been used high levels of brood mortality were observed on frames which had been bumped, which had gone unreported in previous similar studies (Fig 2).

The NBU monitoring of ESA apiaries for *Tropilaelaps* spp. now utilises a combination of both brood uncapping and sugar roll techniques, with both techniques being carried out on 50% of the colonies or a minimum of four colonies in an apiary. The impact of any surveillance technique on a colony and the practicalities of carrying out any surveillance method in the field in ‘real life’ scenarios should be an important consideration when determining a techniques usefulness. Issues such as brood mortality due to the bump method and the refinement to the brood uncapping technique which were observed during this study where valuable outcomes from this research as determining what doesn’t work in any given situation is as valuable as determining what does. This study has refined and improved existing detection techniques for *Tropilaelaps*, discounted ineffective and damaging detection techniques and given government agencies and beekeepers alike a new, reliable, rapid, non-destructive and cheap surveillance technique.

## Supporting information

S1 File Raw data file. (XLSX)
