# Correction: Assessment of the efficacy of field and laboratory methods for the detection of *Tropilaelaps* spp.

**DOI:** 10.1371/journal.pone.0316864

**Published:** 2024-12-30

**Authors:** The PLOS ONE Staff

## Notice of Republication

An incorrect, earlier version of this article was published in error. The publisher apologises for the error. This article was republished on December 18, 2024, to correct for this error. Please download the article again to view the correct version. The originally published, uncorrected article and the republished, corrected article are provided here for reference.Reference

## Supporting information

S1 File Originally published, uncorrected article. (PDF)

S2 File Republished, corrected article. (PDF)
